# Effect of Moisture Exchange on Interface Formation in the Repair System Studied by X-ray Absorption

**DOI:** 10.3390/ma9010002

**Published:** 2015-12-22

**Authors:** Mladena Lukovic, Guang Ye

**Affiliations:** Section of Materials and Environment, Faculty of Civil Engineering and Geosciences, Delft University of Technology, Delft 2628 CD, The Netherlands; g.ye@tudelft.nl

**Keywords:** moisture exchange, repair system, interface, void content

## Abstract

In concrete repair systems, material properties of the repair material and the interface are greatly influenced by the moisture exchange between the repair material and the substrate. If the substrate is dry, it can absorb water from the repair material and reduce its effective water-to-cement ratio (w/c). This further affects the hydration rate of cement based material. In addition to the change in hydration rate, void content at the interface between the two materials is also affected. In this research, the influence of moisture exchange on the void content in the repair system as a function of initial saturation level of the substrate is investigated. Repair systems with varying level of substrate saturation are made. Moisture exchange in these repair systems as a function of time is monitored by the X-ray absorption technique. After a specified curing age (3 d), the internal microstructure of the repair systems was captured by micro-computed X-ray tomography (CT-scanning). From reconstructed images, different phases in the repair system (repair material, substrate, voids) can be distinguished. In order to quantify the void content, voids were thresholded and their percentage was calculated. It was found that significantly more voids form when the substrate is dry prior to application of the repair material. Air, initially filling voids and pores of the dry substrate, is being released due to the moisture exchange. As a result, air voids remain entrapped in the repair material close to the interface. These voids are found to form as a continuation of pre-existing surface voids in the substrate. Knowledge about moisture exchange and its effects provides engineers with the basis for recommendations about substrate preconditioning in practice.

## 1. Introduction

Moisture transport between a cementitious repair material and a concrete (or mortar) substrate determines the microstructural development of the interface and repair material in concrete repair systems [1,2]. Still, this area of research remains scarcely understood, because the dynamics of water exchange is very complicated and strongly influenced by hydration of the repair material. Only a few studies on the moisture exchange in multilayer systems when fresh, newly-cast material was placed on the matured substrate have been reported [3,4,5]. In all of these studies, nuclear magnetic resonance (NMR) technique was used. Some preliminary studies on layered “Lego blocks” specimens made of freshly cast cement pastes were performed by using X-ray absorption [6]. Most of these studies only focused on the moisture exchange: they indicated the relative change of moisture content in the repair system. Effects of the moisture exchange on the microstructure of the interface and repair material were not studied. Interface microstructure between a brick and mortar after moisture movement was only investigated by Brocken *et al.* [3].

The aim of this paper is to study effects of the moisture exchange and the substrate preparation on the interface and repair material formation in a repair system. The X-ray absorption technique is first used to quantitatively study moisture movement in a repair system. Ordinary Portland cement (OPC) paste with w/c of 0.3 is used as a repair material. Samples were sealed for 3 d and during this period the moisture exchange was monitored. Subsequently, the internal microstructure of the repair system is studied by CT scanning in order to investigate the consequences of moisture exchange on the formed structure.

## 2. Materials and Methods

### 2.1. Materials and Sample Prepartion

**Figure 1:**
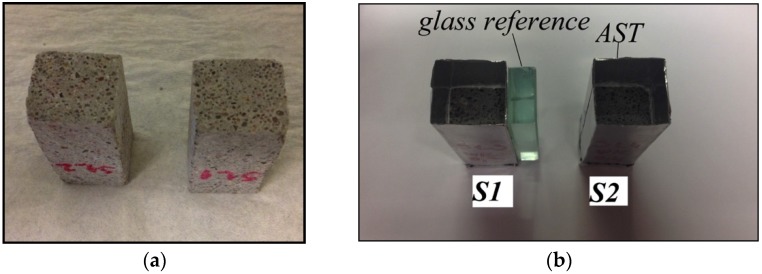
Preparation of the mortar substrate sample: (**a**) After cutting and polishing; (**b**) In a mold and covered by aluminum self-adhesive tape (AST).

The substrate used in the study was a two year old mortar. A standard mortar mixture (OPC CEM I 42.5N (ENCI, Rotterdam, The Netherlands), w/c 1:2, cement-to-sand ratio 1:3) was used. After two years of fog curing (100% relative humidity, temperature 20 °C), two small prism specimens (18 × 19 × 40 mm^3^) were cut with a diamond saw from bigger mortar samples. Prior to casting of the repair material, top surface of the substrate was polished to minimize the influence of surface roughness (Figure 1a). Two samples (marked as S1 and S2) with a sealant on the sides were then placed in a mold and covered with aluminum self-adhesive film (AST) to prevent water evaporation from the sides (Figure 1b). A glass reference was placed between two samples in order to account for variations in the beam intensity, as later explained.

Before covering with AST, mortar substrate was preconditioned in two ways. Dry substrate (DS) simple was dried in an oven at 105 °C until constant weight was achieved. This was done in order to remove all evaporable water and create a zero initial moisture content at the beginning of the experiment [7]. It has to be noted that these conditions might trigger some microstructural changes and microcracking in the material [8]. The other mortar substrate was kept in the fog room prior to casting of the repair material. This substrate was considered as wet (saturated) substrate (WS). For repair materials, cement paste with OPC CEM 42.5 N and w/c of 0.3 was used.

### 2.2. X-ray Absorption for Moisture Content Measurements

**Figure 2:**
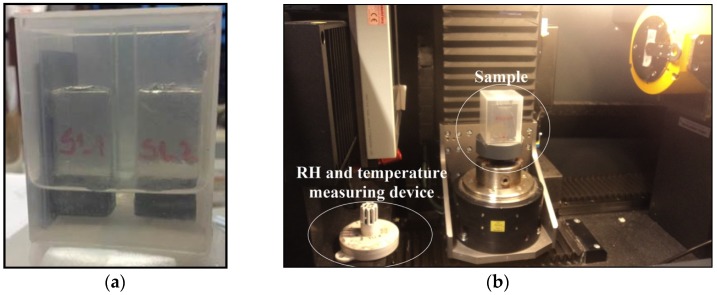
Samples for moisture exchange measurements; (**a**) two specimens are placed in a plastic container prior to X-ray testing; and (**b**) the X-ray system with the position of the sample and temperature and RH sensors.

After the preparation, two samples and a glass reference were placed in a plastic container (Figure 2a). Subsequently, container is placed in a Phoenix Nanotom X-ray system (Phoenix|x-ray, Wunstorf, Germany, Figure 2b) where water exchange was measured. The apparatus is equipped for Computer Tomography (CT scanning), but in this part of the study was limited to X-ray imaging without specimen rotation. A comparison between two X-ray images taken at the beginning and after a certain time step provides a moisture change in the sample at a certain time step. Even though relative humidity (RH) and temperature (T) are not controlled in the X-ray system, RH and temperature measuring devices (see in Figure 2b) were placed in an X-ray system chamber during testing.

When an object is irradiated with an X-ray, the X-ray is attenuated (scattered and absorbed) due to the interaction with the material. The attenuation behavior of monochromatic X-rays (X-ray photons of a single, consistent energy) can be described by the Beer-Lambert law [9]:

(1) $$I=I_{0}e^{-\mu d}$$

where *µ* is the attenuation coefficient; *d* is the thickness of the sample; and *I_0_* is the incident intensity. An attenuated X-ray results in transmitted intensity *I*. Detector visualizes intensities levels as grey scale values (GSV) [10]. Therefore, the attenuation coefficient of material with a known thickness can be determined by knowing the change in intensity level, *ln(I/I_0_)* (Equation (1)), or by knowing the change in GSV, *ln(GSV/GSV_0_)*.

**Figure 3:**
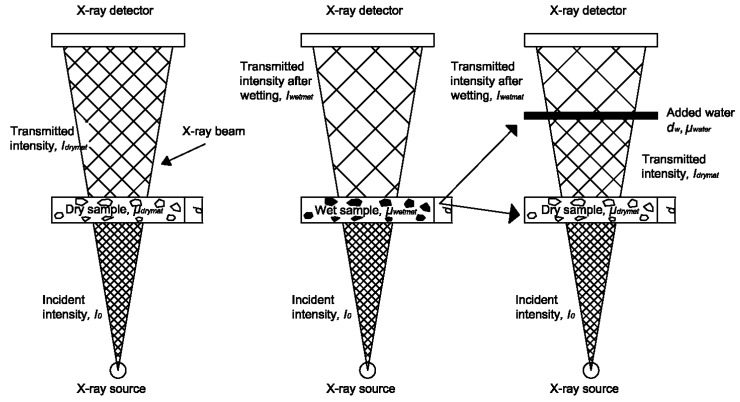
The moisture distribution is obtained by logarithmically subtracting an image of the dry sample *I_dry_* from the image of the wet sample *I_wet_*, adopted from Reference [10].
- Once the attenuation coefficient of water is determined, GSV of the wet porous material can be correlated to GSV of the dry porous material according to the following equation (also derived from Equation (1)):
  (3) $${GSV}_{wetmat}={GSV}_{drymat}e^{-\mu_{water}d_{w}}$$
  where *d_w_* is the thickness of fictitious water layer equivalent to the additional moisture content in sample (see Figure 3) and can be expressed as:
  (4) $$d_{w}=\Delta c_{water}\frac{d}{\rho_{water}}$$
  Here, *Δc_water_* is the change of water content (g/cm^3^); *d* is the thickness of the sample; and *ρ_w_* is the density of water. From these equations, *Δc_water_* can be determined as:
  (5) $$\Delta c_{water}=-\frac{\rho_{water}}{\mu_{water}d}\text{ln}\frac{{GSV}_{wetmat}}{{GSV}_{drymat}}$$
- In this study, only the middle part of the specimen (around 16 mm) is analyzed in order to exclude the influence of edges (see Figure 5). Obtained moisture profiles are then averaged over the specimen’s width. As a result, the change in moisture content is obtained as function of specimen height.

During moisture transport (either wetting or drying), GSV of material is changing. Correlating GSV change with the change in moisture content is done by making use of a simple physical principle. In a dry sample, X-rays are attenuated by the dry material only (Figure 3-left). By scanning through the dry material, a reference image is obtained. If water is added to the porous material, the attenuating material will consist of the dry material plus a thickness of a fictitious water layer (*d_w_*), equivalent to the additional moisture content of the material (Figure 3-right) [10]. Additional moisture content is obtained by logarithmically subtracting a reference image from the image taken during moisture exchange. Therefore, provided that the change in beam intensity, *ln(I/I_0_)*, and attenuation coefficient of water are known, the (additional) moisture content inside the material can be determined according to Equation (1). In this case *I_0_* corresponds to beam intensity after passing through the dry material, while *I* corresponds to beam intensity after passing through the wet material. For quantification of the moisture content change, the following procedure was used.

**Figure 4:**
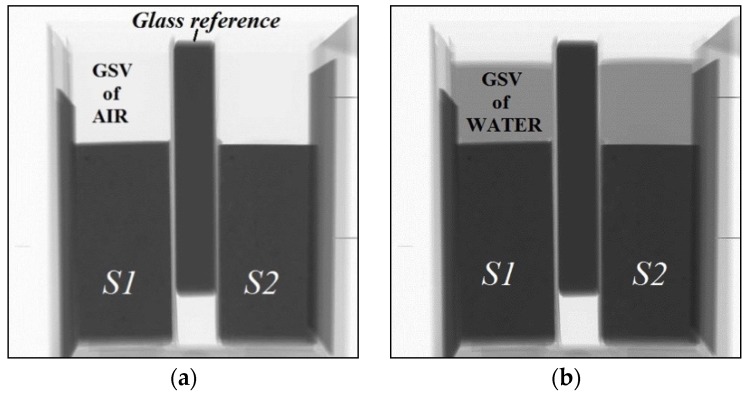
Difference in GSV between an empty and water filled container placed at the top of the samples S1 and S2, used for calculating attenuation coefficient of water: (**a**) GSV of an empty container; and (**b**) GSV of water-filled container.

- The attenuation coefficient of water can be determined based on the GSV change of empty and water filled container with a known thickness (see Figure 4). The following formula can be used:
  (2) $${GSV}_{water}={GSV}_{air}e^{-\mu_{water}d_{water}}$$
  where *GSV_water_* is the greyscale value of water; *GSV_air_* the grey scale value of air; *d_water_* is the thickness of water (which is equal to the thickness of the container); and *µ_water_* is the attenuation coefficient of water. The first image is made with a beam passing through an empty container and *GSV_air_* (corresponds to *I_0_*) is obtained (Figure 4a). The second image is made with a container filled with water and *GSV_water_* (corresponds to *I*) is obtained (Figure 4b). Thus, the unknown *µ_water_* can be calculated based on Equation (2). Note that this equation is equivalent to Equation (1). Further discussion about the attenuation coefficient of water when polychromatic X-ray is used, is given in the following subsection.

Due to slight variations in the beam intensity, the obtained GSV varies even without a change in the moisture content. In order to account for this effect, a glass reference was used in all analyses (see Figure 1b and Figure 4a). It is considered that glass does not absorb water and therefore, in this region, GSV should be constant during the analysis. Therefore, each image was normalized according to the variations of GSV of the glass reference in order to account for changes in the beam intensity.

The parameters for X-ray analysis were set as: X-ray tube voltage 130 kV, X-ray tube current 270 µA. The spatial resolution was 30 µm/pixel. Each image used in the analysis (a representative image) is an average of 25 images. With 0.5 s needed for acquisition of an image, a representative image for a certain time step was obtained in 12.5 s.

**Figure 5:**
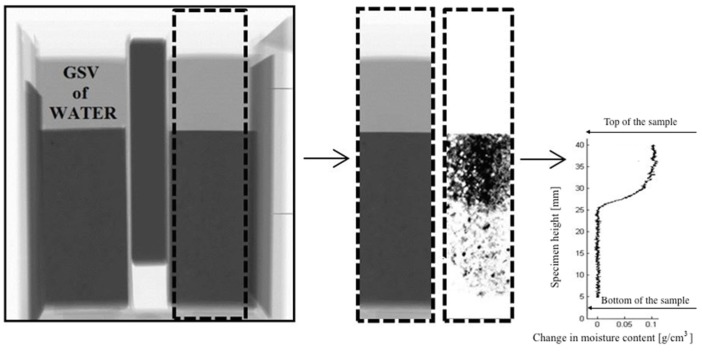
An example of the original X-ray image and analyzed part of the specimen from which the moisture profile is calculated.

#### 2.2.1. Determination of the Attenuation Coefficient

When using polychromatic X-ray source (photons emitted over a spectrum of energy levels), such as the one used in this research, two aspects should be considered in order to determine the attenuation coefficient of water [11].

**Figure 6:**
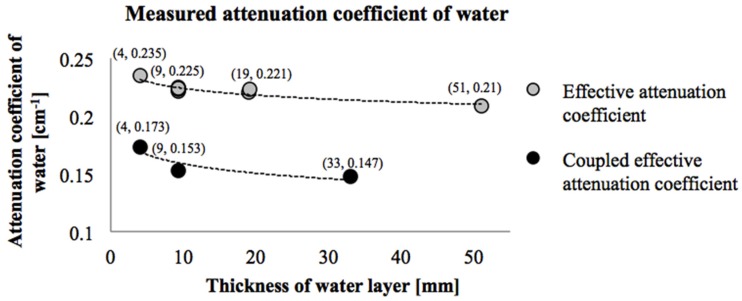
Influence of water layer thickness and parent material (mortar substrate) on measured attenuation coefficient of water.

The first aspect is that the attenuation coefficient of water depends on the water layer thickness. This dependency is a consequence of using polychromatic X-ray source which leads to so called “beam hardening” effect: while photons are passing through the material, lower energy X-ray photons are attenuated easier, the energy spectrum is changing, and progressively, with increasing thickness of material, remaining protons become “harder” to attenuate. As a result, the measured attenuation coefficient will depend on the thickness of the material. Attenuation provided by a certain thickness of a material is described by the term “effective attenuation coefficient”. Pease *et al.* [11] observed that, with the increasing thickness of the water (and also other materials such as clay brick, concrete, and wood), the effective attenuation coefficient of material decreases. The effective attenuation coefficient of water in this research, measured for the different water layer thickness (Figure 6), shows the same behavior.

The second aspect is related to the beam-hardening effect and the widely used non interacting composite system (Figure 3), which assumes that the porous parent material does not influence the attenuation coefficient provided by water. In the absorption (or drying) tests, however, photons are attenuated both by the parent material and the water. Therefore, the attenuation coefficient of water cannot be determined independently of the parent material (in this case mortar substrate). In order to account for this, Pease *et al.* [11] introduced the term called "coupled effective attenuation coefficient" of water which is a function of the parent material and its thickness. They observed that, with increasing thickness of different materials (e.g., concrete, cement paste, calcium silicate, *etc*), the coupled effective attenuation coefficient of water decreases. In this research, the same was tested by placing the 18 mm thick mortar substrate in front of the water holder (with inner dimensions of 4 mm, 9 mm, and 33 mm). The obtained coupled effective attenuation coefficient of water was lower than the effective one (Figure 6), similar to findings of Pease *et al.* [11]. 

As the thickness of the water layer should be similar to the anticipated maximum change of the water content in the parent material, the attenuation coefficient of the 4 mm thick water layer, placed behind the 18 mm thick mortar substrate (0.1728 cm^−1^) was used further to quantify the change of the water content. Accordingly, the maximum change of the water content in the mortar substrate is assumed to be around 0.22 g/cm^3^, which should be reasonably close to the maximum moisture content of the mortar.

#### 2.2.2. Limitations of the Experiment 

**Figure 7:**
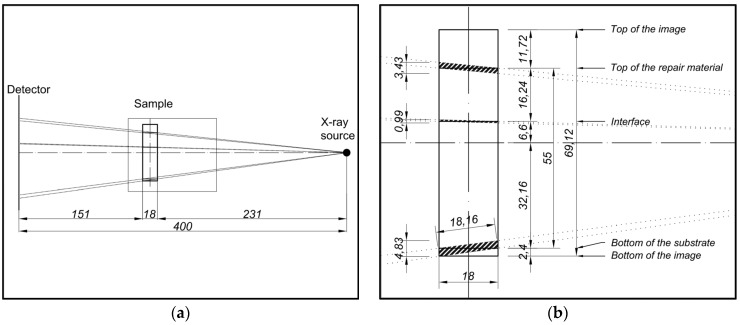
Experimental setup and influence of the conical beam on the measured data, units are in (mm): (**a**) Position of the detector, the specimen and the source; (**b**) Beams passing through the sample, high magnification.

An important limitation of this technique is caused by the conical beam of the X-ray setup. As a result, the thickness of the material through which the beam passes depends on the position of the source with respect to the specific location (*i.e.*, coordinate) in the sample (Figure 7a). Consequently, at the top and the bottom of the sample, the thickness of the attenuating material is larger, and this will affect the effective attenuation coefficient. The thickness of the sample through which the beam is passing varies between 18 mm in the middle of the specimen (*i.e.*, when the beam is perpendicular to the specimen) and 18.16 mm at the top and bottom (Figure 7). In the current research, this was not taken into account, but should be considered for improvement of the technique.

One more consequence of the conical beam is that X-ray photons are not emitted parallel to the repair/substrate interface. The interface location, therefore, is not well defined, and it includes a small zone of both the repair material and the substrate. The scattering of the X-ray beam is, therefore, modified in this zone. The width of this zone was estimated to be around 0.99 mm (Figure 7). This interfacial zone is marked further in the chapter with the dashed line (of the similar thickness) to indicate the unreliability of the results. Similarly, the bottom and the top of the specimen are also affected (4.83 mm and 3.43 mm, respectively, see Figure 7), and these parts were excluded from the graphs. Note that these effects are dependent on the specimen thickness and the distance between the object and the source. 

### 2.3. Degree of Hydration Measurements 

Degree of hydration of tested repair materials was measured based on non-evaporable water content and according to the procedure given in [12]. Repair materials were split from the substrates immediately after 3 d of sealed curing. Then, the samples were ground into powder. The powder was dried in an oven at a temperature of 105 °C for 24 h to remove first the evaporable water. Simultaneously, the crucibles were dried in a furnace at a temperature of 1000 °C. Around 2 g of powder was added to the crucibles and the weight was recorded as *W_105_*. The crucibles were placed in a furnace at a temperature of 1000 °C and left there for 3 h to remove the non-evaporable water. The weight of the sample was measured and recorded as *W_1000_*. Two parallel measurements were done. The degree of hydration, *α*, was calculated using the following equation [13]:

(6) $$\alpha =\frac{W_{105}-W_{1000}}{0.23\times W_{1000}}$$

Equation (6) is simplified as it does not take into account cement loss on ignition [14] and assumes that the hydration of 1 g of anhydrous cement produces 0.23 g of non-evaporable water. These simplifications, however, are considered not to have significant influence on results, as long as raw materials and the assumptions were the same throughout all tests. Note that the measured degree of hydration corresponds to the bulk degree of hydration in the repair material. This means that microstructural differences across the repair material thickness as appear in the practice are not captured.

### 2.4. CT Scanning for the Mesostructure Characterization

After the moisture content measurements (after 3 d of sealed curing), the void content in the repair system was characterized. Tested repair systems were investigated by a CT scanner in order to study effects of moisture exchange on repair system microstructure. During each scan, multiple X-ray images of a specimen are taken at different angles (*i.e.*, 1440 tomographic images were taken over a complete 360° rotation). A single scan took about 90 m to perform. Using a reconstruction algorithm, a 3D image of the internal structure of a specimen is produced. Voxel size was also 30 µm (the same as pixel resolution for measurement of moisture transport).

## 3. Moisture Movement in the Repair System and Discussion

**Figure 8:**
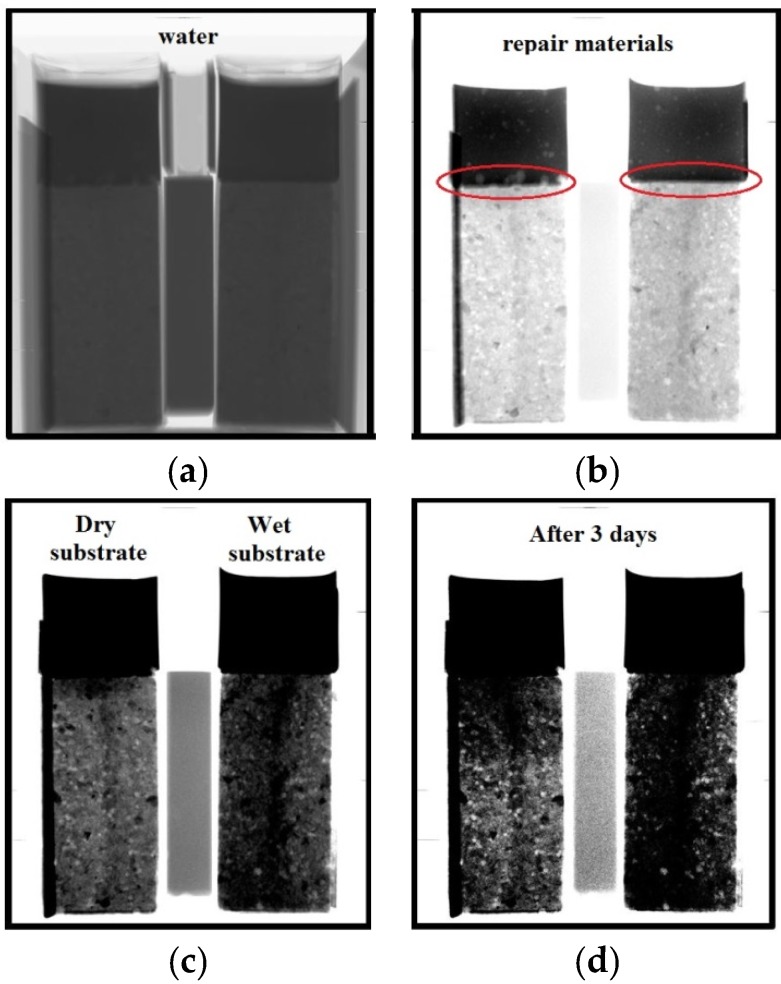
X-ray measurements on repair systems (w/c = 0.3, DS, S3 and w/c = 0.3, WS, S3): (**a**) Reference image; (**b**) Chosen GSV range with the emphasis on the repair material; (**c**) Chosen GSV range with the emphasis on the mortar substrate; and (**d**) Image after 3 d of sealed curing.

Cement pastes with a w/c of 0.3 and around 15 mm thickness were cast on the top of the mortar substrates. As already explained, substrates were preconditioned in different ways. In one case, substrate was wet (specimen marked as w/c = 0.3, WS, S3, Figure 8-left specimen) and in the other, substrate was completely dry (specimen marked as w/c=0.3, DS, S3, Figure 8-right specimen). Immediately after casting, repair systems are sealed with aluminum self-adhesive tape and placed in an X-ray system in order to investigate the moisture exchange between repair materials and mortar substrates (Figure 8). Temperature and relative humidity were recorded during the experiment. Average temperature and relative humidity were 27.69 °C and 16.83% with standard deviations of 0.49 °C and 1.18%, respectively.

It is assumed that the main component absorbed by the mortar substrate from the repair material is water. Therefore, although cement slurry (and not the pure water) is actually absorbed by the mortar substrate, the coupled effective attenuation coefficient of water is used in the calculations.

**Figure 9:**
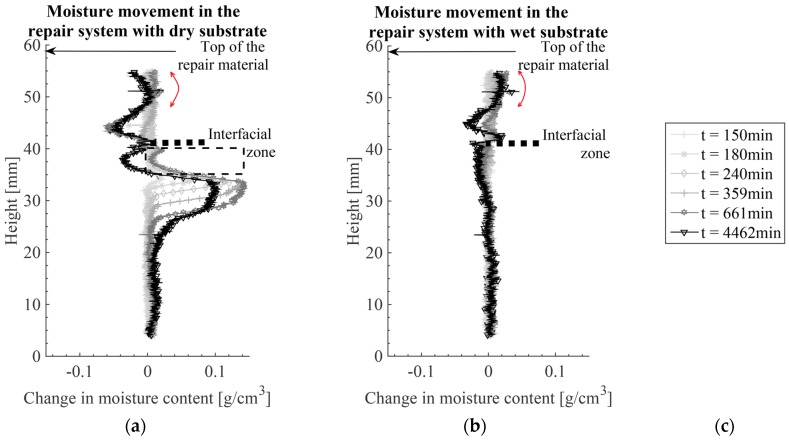
Moisture profiles in the repair systems as a function of time, *t* (*t* after mixing the repair material): (**a**) dry substrate, w/c = 0.3, DS, S3; (**b**) wet substrate, w/c = 0.3, WS, S3 with the legend; and (**c**) legend.

**Figure 10:**
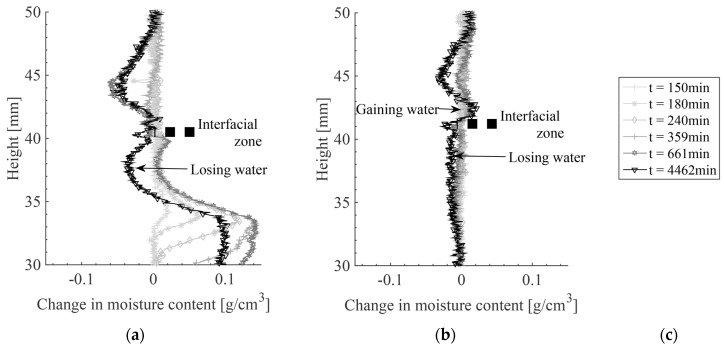
Moisture profiles in the repair systems close to the interface (dashed line): (**a**) dry substrate, w/c = 0.3, DS, S3; (**b**) wet substrate, w/c = 0.3, WS, S3; and (**c**) legend.

The reference image was made 136 min after mixing the repair material. The first measured profile is obtained 14 min after making the reference image and 150 min after mixing the repair material (Figure 9 and magnified moisture profiles in Figure 10). As a consequence, the change in moisture profiles at the top of the substrate (close to the interface) could not be captured. These parts (indicated by a dashed rectangle in Figure 9a) are filled with water during the initial 150 min. As previously discussed, the interface is not parallel to the X-ray beam and, therefore, the scattering process is modified in this zone. Therefore, the results in this zone are not reliable and are indicated by the dashed line. As in first 5 h, moisture absorption is constant and consistent (see for more details [15]), the test was continued.

In order to perform the integration procedure and calculate the cumulative water content for the whole repair system, three integration limits are defined: top of repair material (defined as the repair material height immediately after casting, top arrow in Figure 9), interface (dashed line), and bottom of the mortar substrate (where the moisture profiles begin). In these calculations it is assumed that the coupled effective attenuation coefficient of the water in the repair material is equal to the coupled effective attenuation coefficient of the water in the mortar substrate. In Figure 6 it was shown, however, that the coupled effective attenuation coefficient is sensitive to the porous material thickness and the thickness of water layer. These are probably not the same for the repair material with w/c of 0.3 (*i.e.*, 23% of water and 77% of OPC by mass of the paste) and the mortar substrate with w/c of 0.5 (*i.e.*, 66% of aggregates, 11% of water, and 22% of OPC by mass of mortar). Furthermore, the calculation of the water movement within the repair material is very complex because of the ongoing hydration and the volume changes in the material. The height of the repair material is changing due to the setting in the early stage and later due to shrinkage. In addition, the use of a conical X-ray beam introduces the difficulties in defining the exact location of the interface (as previously explained). Having in mind all these assumptions and simplifications, further calculations for the water content inside the repair material should be taken only as indicative.

**Figure 11:**
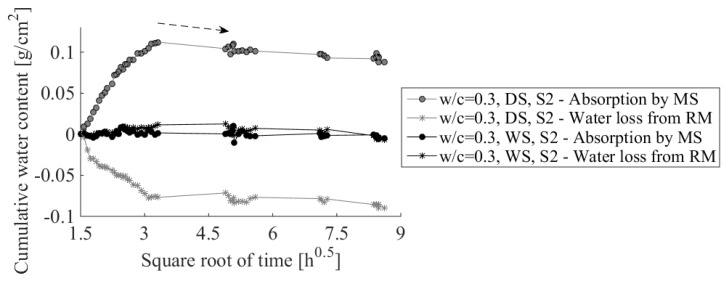
Calculated cumulative water contents in w/c = 0.3, DS, S2 and w/c = 0.3, DS, S3. MS denotes mortar substrate and RM denotes repair material.

The cumulative water content as a function of time is calculated for the repair material and the substrate and curves are given in Figure 11. The dry substrate is absorbing water (cumulative water content is positive) while the repair material is losing water (cumulative water content is negative). If the calculation procedure and sealing are perfect the amount of water that is lost from the repair material should be equal to that absorbed by the substrate. However, amount of water absorbed by the substrate is higher. As both aluminum foil and sealant are used for sealing the specimen, it is considered that sealing is good but that numerous assumptions in the calculation (as previously explained), led to differences in calculated cumulative water contents. 

Contrary to the repair material cast on initially dry substrate in w/c = 0.3, DS, S3, the repair material was cast on saturated substrate in w/c=0.3, WS, S3 did not lose water. This is because the substrate was saturated and could not absorb the water from the repair material. Although the repair material is hydrating (some water is becoming chemically bound), the mass of water inside the material is not changing. Therefore, only moisture movement, and not hydration, is monitored with X-ray absorption. Although hydration was not captured, moisture redistribution within the repair material itself (as a consequence of hydration) was. Moisture is moving from the interface zone to the bulk repair material. The interface region (30–50 μm) is typically characterized by a locally higher w/c (less cement) than the bulk repair material. Due to the coarser pore structure, water from this porous region will be taken to the denser bulk paste, resulting in creation of large empty pores within the interface, as explained by Bentz and Hansen for aggregate-paste ITZ [6]. As a result, moisture profiles are becoming concave (indicated by arrows in Figure 9a,b). The same phenomenon is also modelled with hydration model [16]. 

In a repair system, however, some water for hydration of the interface can be partially taken back from the saturated substrate. Negative shift in cumulative moisture content in Figure 11 (marked with the dashed arrow), indicates moisture movement from the substrate to the repair material. However, water loss in this region (indicated in magnified profiles in Figure 10a) can also be a consequence of water redistribution in the substrate itself, as the moisture profile in the repair material does not clearly confirm this. Although from the moisture profiles water gain in the repair material cannot be clearly seen, it should exist. This is because of the hydration of the repair material and water redistribution in the repair system in order to again reach hygral equilibrium. In the repair material, there are two causes of water loss: water taken by the unsaturated substrate and hydration reaction of the material itself (self-desiccation). Hydration of the repair material causes the consumption of capillary water from the repair material, thereby reducing the relative humidity that, at a certain point in time, was in equilibrium with the substrate. Therefore, a new capillary pressure potential difference is created. In order to restore the equilibrium state, water is driven back from the substrate to the repair material (w/c = 0.3, DS, S3, indicated by arrow in Figure 11). In sample w/c = 0.3, WS, S3 (Figure 10b) this can be clearly seen. The substrate is losing water from the top while the moisture profiles in the repair material indicate water gain close to the interface. In this sample, therefore, moisture profiles both in the substrate and the repair material confirm the moisture exchange.

## 4. Effects of Moisture Movement in the Repair System and Discussion

### 4.1. Effect of Moisture Movement on the Degree of Hydration of the Repair Material

**Figure 12:**
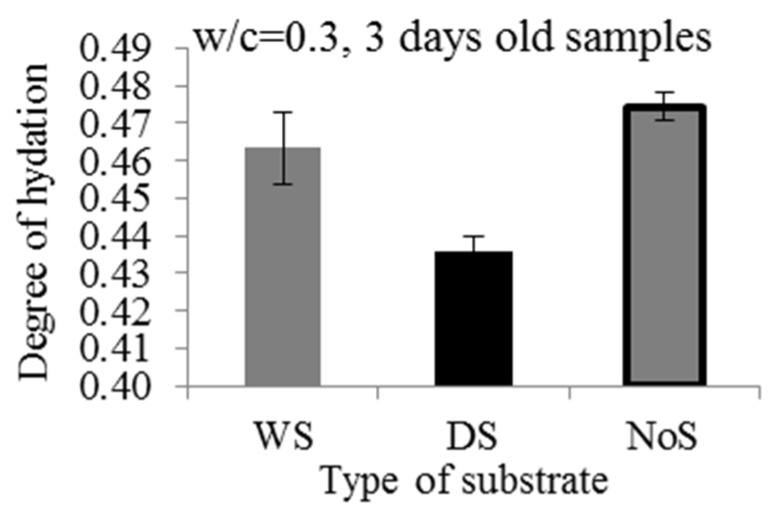
Influence of the mortar substrate saturation on the measured degree of hydration of the repair material, w/c = 0.3, WS, S2 (WS), w/c = 0.3, DS, S3 (DS) and bulk repair material (NoS).

After 3 d, samples w/c = 0.3, DS, S3 and w/c = 0.3, WS, S3 were taken out of the X-ray system. Repair materials were split from the substrates and degree of hydration was measured (75 h after casting). Apart from repair materials that were cast on top of the substrates (DS and WS), bulk repair material was cast in a separate mold (NoS) and placed in the same conditions as repair systems (inside the X-ray system chamber), was also tested. Degrees of hydration for all three samples and standard deviations are given in Figure 12.

Repair material cast on a wet substrate has a lower degree of hydration compared to the bulk repair material (NoS in Figure 12). A possible reason is the sealing procedure. The bulk repair material mold (NoS) was easily closed with the lid immediately after casting. On the other hand, with the repair material cast at the top of the substrate, this sealing took some time (20 m), meaning that during this period water could evaporate. Taking into account standard deviation, differences in measured degrees of hydration in these two samples (with NoS and WS) were not big.

Dry substrate caused a reduction in the degree of hydration of the repair material. This is due to the reduction of the effective w/c caused by moisture loss of the repair material. This reduction would be significantly higher with the higher absorption rates of the substrate. Furthermore, with thinner layer of repair material, the reduction would be probably even higher. This is because the substrate needs less time to absorb water from the repair material. As a result, hydration will be hindered or stopped even earlier.

### 4.2. Effect of Moisture Movement on Microstructure of the Repair Material and Interface

**Figure 13:**
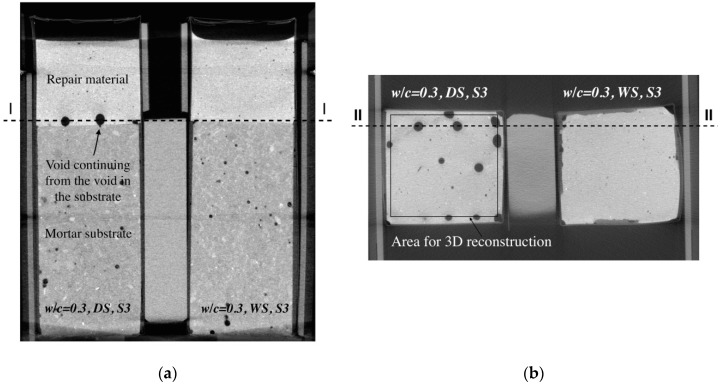
CT scan images for specimens w/c = 0.3, DS, S3 and w/c = 0.3, WS, S3 made at the age of 3 d: (**a**) section II-II (Figure b), front view; and (**b**) section I-I (Figure a), top view.

**Figure 14:**
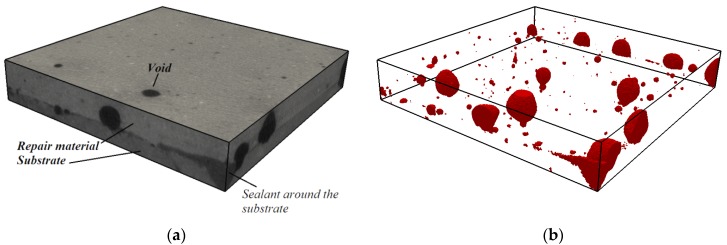
Void content in the repair system close to the interface, w/c = 0.3, DS, S3 (3mm thickness): (**a**) original structure (whole GSV range); and (**b**) thresholded voids (chosen GSV range).

**Figure 15:**
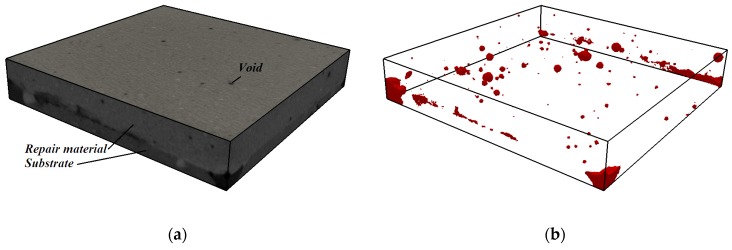
Void content in the repair system close to the interface, w/c = 0.3, WS, S2 (3mm thickness): (**a**) original structure (whole GSV range); and (**b**) thresholded voids (chosen GSV range).

After measurements of the moisture transfer, the specimens were scanned in a CT scanner to investigate the effects of moisture exchange on resulting microstructure of the repair system (resolution is 30 µm). Two cross sections are given in Figure 13. Section I-I is made directly above the interface in two specimens (w/c = 0.3, DS, S3 and w/c = 0.3, WS, S3). There is a clear difference in void content in the two specimens. In order to investigate this further in 3D, all the slices parallel to the interface are aligned on top of each other. 3D structure is made and images of the scanned system close to the interface (containing around 1.5 mm of the substrate and 1.5 mm of the material) are given in Figure 14 and Figure 15. The repair material can be clearly differentiated from the substrate. By segmenting (thresholding) the dark pixels (representing voids) in the original image, the image showing only voids in the repair system can be obtained (Figure 14b and Figure 15b). A comparison between the original 3D image (whole GSV range) and the thresholded image (chosen GSV range) is given in Figure 14a,b. As the greyscale value of the void and the sealant around the mortar substrate are very close to each other, the sealant can be also observed in thresholded images (Figure 14b and Figure 15b).

**Figure 16:**
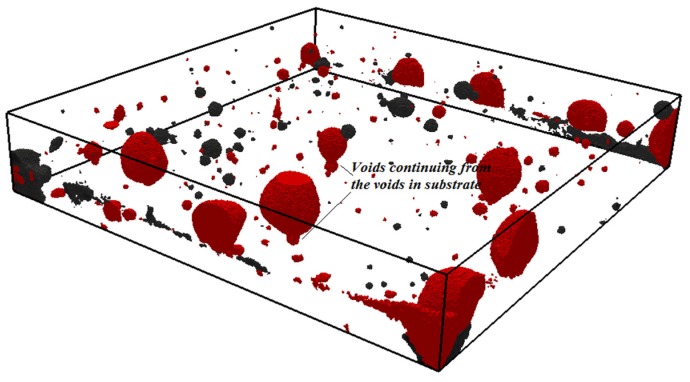
Void content in the repair system close to the interface w/c = 0.3, WS, S2 and w/c = 0.3, DS, S3, influence of the substrate saturation (black if the substrate is wet, red if the substrate is dry).

The moisture exchange between the repair material and substrate seems to have a significant influence not only on the effective w/c, degree of hydration and uniformity of properties of the repair material and interface, but also on void content close to the interface. The void content in two samples is compared in Figure 16. Significantly more voids are observed when the substrate is dry. Dry substrate absorbs water from the repair material (Figure 9a). When the water is absorbed, air from the substrate is being released. This air stays entrapped at the interface resulting in high void content. 

It was also observed that voids from the substrate are “initiation” points for voids in the repair material (Figure 13a and Figure 16). A void from the substrate is continuing in the repair material, making this an intrinsically weaker zone. In this system, the mortar substrate was a well-cured specimen with low porosity. It is anticipated that, with a more porous substrate, this influence would be significantly higher. In addition, it would be interesting to investigate if the same would be observed around the microcracks or cracks that are usually present in a substrate. In substrates used in this research, with a resolution of 30 µm, no cracks were observed. 

**Figure 17:**
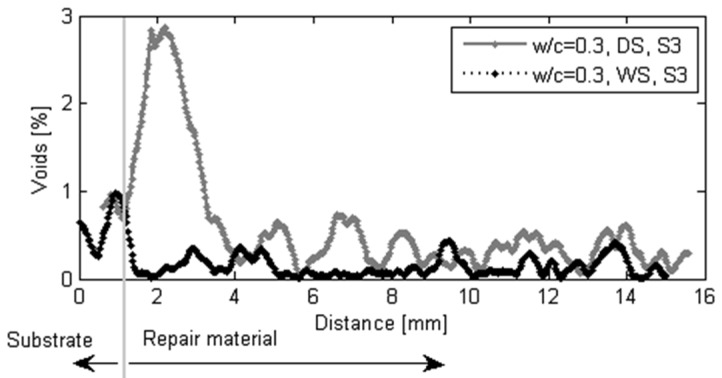
Void content in the repair system close to the interface (influence of the substrate saturation).

The void content close to the contact of two materials can be also quantified. In order to calculate the void content, an area of interest is chosen inside the images (Figure 13b). Every image, starting from the substrate approximately 1 mm below the interface, is analyzed. The percentage of voids in thresholded images is calculated by dividing the void area (number of red pixels in Figure 14b and Figure 15b) by the total area (all pixels). Spacing between the images is 30 µm. As a result, void content as a function of distance from the interface is obtained. In w/c = 0.3, DS, S3, 2.5 mm of the repair material closest to the interface is affected by moisture absorption and result in higher volume content compared to the repair material cast on the fully saturated substrate (Figure 17).

High content of air voids at the interface of the cement paste cast on initially dry brick was also observed in the study of Brocken *et al.* [3]. They analyzed the whole-section specimen and concluded that only 20%–30% of surface area of the paste is in good contact with the fired-clay brick. With sand-lime brick they found a better bond: 55%–70%. They suggested that poor bond and high void content might be attributed to “ineffective contact of the cement paste during laying on the brick: presumably due to fast compaction and poor laying”. Another reason might be the phenomenon observed in this research: the brick is absorbing water, hereby releasing air which stays entrapped at the interface.

**Figure 18:**
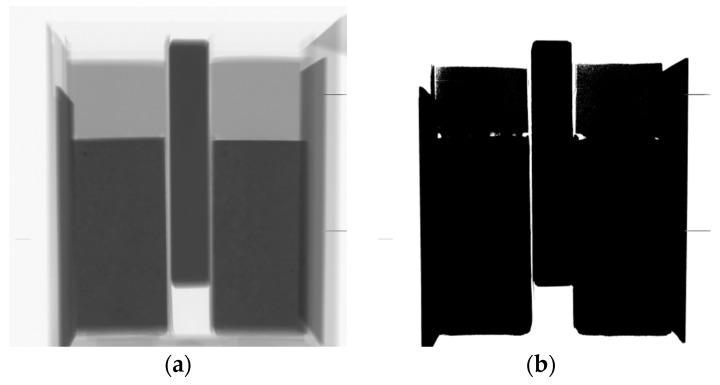
Images from X-ray absorption measurement when water is absorbed by the substrate: (**a**) original; and (**b**) thresholded image.

**Figure 19:**
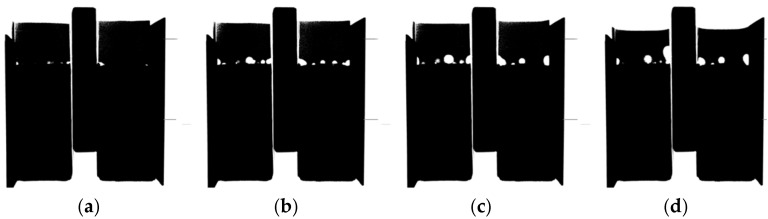
Void development when water is absorbed by the mortar substrate: (**a**) Reference image; (**b**) After 7 min; (**c**) After 17 min; (**d**) After 240 min.

Air release from the substrate due to the moisture movement is not only related to the repair material. It is also observed when water is being absorbed by the substrate (Figure 18 and Figure 19). Original X-ray images were thresholded so that voids can be easily observed (Figure 18). In Figure 19, air void development at the surface of mortar substrate as a function of time is given. With time, void content in the contact zone between water and mortar is also increasing. 

This was not previously reported for the repair system performance (or any multilayer system with porous materials). However, similar performance as in repair systems is observed in light weight concretes [17]: ”Water absorption of the light weight aggregates (LWA) after the concrete leaves the mixer, is accompanied by an expulsion of air from the particles. This air will form a rim of bubbles at the LWA surface. For some types of LWA, this amount of expelled air during setting is considerable and the aggregate-paste bond might be weakened. This is particularly the case when using LWA with a medium water absorption, that means neither very open capillary and fast absorption nor very impermeable. A normal procedure to repair this damage is remixing the concrete after a period and thus getting the bubbles evenly distributed in the paste.”

“Medium water absorption (neither impermeable nor very open capillary)” is probably the condition to be expected for most of the concretes that should be repaired. The only practical solution is saturating concrete substrate prior to repair application. For practical application, it is important to know how long the substrate should be saturated prior to casting of the repair material. It seems important, therefore, to investigate whether the void content at the interface would differ with different saturation regimes of the substrate.

If the substrate is not saturated, water will be absorbed from the repair material. As a result, effective w/c of the repair material will be reduced. Therefore, bond strength due to lower resulting w/c, can be higher compared to the repair system with fully saturated substrate, as previously reported [1]. However, avoiding to pre-saturate the substrate in order to enable higher interlocking and possibly higher bond strength is probably not a good approach. A proper approach would be to directly design a repair material with lower w/c (if needed) and with a pre-saturated substrate with the dry surface. A more uniform and controlled microstructure of the repair material and interface would be obtained. Only then will the repair material reach its designed properties. Otherwise, specifying a certain w/c of repair material is meaningless, because this will change once the repair material is cast. 

Implications of these tests for the reflective cracking phenomenon should be investigated. It might be expected that, once the repair material is cast, if the substrate contains microcracks, these cracks will also absorb water and release air. This air might stay entrapped at the interface, making the region around the existing crack weaker area in the repair material as well. Consequently, this area might be susceptible to further cracking.

## 5. Conclusions

In this paper, X-ray absorption technique was used to investigate the dynamics of moisture exchange in repair systems and its effects on the formed microstructure. Based on the presented study and the investigated parameters, the following conclusions can be drawn:

- Water exchange in a repair system has a critical influence on the microstructure formation of the bulk repair material and the interface. Water loss of the repair material by the substrate absorption reduces the effective w/c and degree of hydration of the repair material.
- The microstructure of the interface is significantly affected by the moisture exchange. Higher absorption of the substrate results in a more porous interface and bulk repair material. Pores and voids in the substrate, which are initially air-filled, are releasing this air to get water. Due to the high viscosity of the repair material and difficulties in compacting, this air remains entrapped at the interface.
- In the repair practice, the substrate should always be pre-saturated with the dry surface. Care should be taken that there is no water layer trapped at the surface of the substrate prior to the application of the material. This will provide a more uniform microstructure development in the repair material and a denser interface. Otherwise, properties of the repair material and the interface will be strongly influenced by the porosity, microcracks, moisture content, and absorption rate of the substrate. This is something that is difficult to control while designing repair systems.

In the future, the X-ray technique presented here can be improved and the same samples should be also tested by different methods (*i.e.*, neutron radiography, NMR) to compare the results. In addition, sensitivity studies of the coupled effective attenuation coefficient for the cement paste and mortar with different w/c and aggregate content should be performed.
